# Aesthetic Attributes of Museum Environmental Experience: A Pilot Study With Children as Visitors

**DOI:** 10.3389/fpsyg.2020.508300

**Published:** 2020-10-19

**Authors:** Claudia Annechini, Elisa Menardo, Rob Hall, Margherita Pasini

**Affiliations:** ^1^Department of Human Science, University of Verona, Verona, Italy; ^2^Environmetrics Pty. Ltd., Killara, NSW, Australia

**Keywords:** environmental psychology, architecture, design, children, museum learning, natural built environment, restorativeness, aesthetics

## Abstract

The research project is a small pilot study of the restorative aspects of museum experience on children; these include the sense of fascination during the visit. Museum environmental awareness was a latecomer to Museum and Visitor studies but is now highly valued. No longer just the “objects” contained in the museum fascinate but also the environment itself becomes an object of fascination. Some authors provide a clear categorization of feelings experienced by the visitor during a museum experience and suggest a framework with four categories of satisfying experience: objective, cognitive, introspective, and social. In designing our study, we began with the definition of museum experience and added a fifth category of “environmental experience.” With this term, we refer to the extent to which the physical environment in and around a museum affects visitors. Indeed, our aim is to analyze the visitor’s stream of feelings and opinions during a museum visit (specifically, the MART—Museum of Modern and Contemporary Art of Trento and Rovereto) to find a proper definition of the aesthetic elements characterizing the “environmental preference.” To do this, we referenced classical and experimental paradigms of Environmental Psychology applied to a museum context and building aesthetic researches, combining qualitative and quantitative approaches. The case study involved 41 children, 20 male and 21 female, from two primary school classes in Rovereto (Italy); the average age was 8.3 years old.

## Introduction

In this paper, we review the development of the ways that the relationship between museums and visitors can be understood. Starting from the definition of the “museum experience,” we underline a quite underdeveloped issue in museum studies, that is the relevance of the museum physical environment, also considering the museum as a restorative environment (Packer and Bond, 2010). The museum experience is changing as a result of the recent interest in the emotional nature of museum visiting; some museums are moving away from formal, didactic models of museum learning toward new models that embrace experimental activities. There is a great deal of curiosity about the emotional interactions between visitors and a museum’s exhibits. In recent years, educational and environmental psychology have underlined the relevance of the attributes of the learning setting, searching for the correlation between students in a given context (Linnenbrink-Garcia and Pekrun, 2011). Subsequently, we describe a small case study, aimed at extending our understanding of the ways in which the nature of a museum building can impact young visitors. In particular, we pay attention to the children’s visiting experience within the museum, investigating the interpretation of the children’s aesthetic experience within the museum environment, during and after museum learning activities.

Museums as we know them today evolved from the so-called “Cabinets of Curiosities” that began appearing in the 1500s. These “cabinets” were typically in the hands of wealthy collectors, and some of the collections formed the base on which a number of important existing museums were created. Over time, publicly accessible collections of objects and other artifacts gained value for their potential role in informing the wider community about culture, history, and science. Initially, the relationship between museums and the community was likely to be one of giving rather than interacting. The museum “improved” the visitor and was a source of authoritative knowledge. In recent decades, there has been a shift in the way the relationship is understood. There has been an increasing recognition of the richness of the ways in which a museum might influence people who interact with it. The museum asserts its public service role and places education at the center of that role. According to the ICOM Statutes, adopted by the 22nd General Assembly in Vienna, Austria, on 24 August, 2007: “A museum is a non-profit, permanent institution in the service of society and its development, open to the public, which acquires, conserves, researches, communicates and exhibits the tangible and intangible heritage of humanity and its environment for the purposes of education, study and enjoyment.”

Therefore, education is universally considered one of the main aims of a museum. “Museum Education can be defined as a set of values, concepts, knowledge and practices aimed at ensuring the visitor’s development; it is a process of acculturation which relies on pedagogical methods, development, fulfillment, and the acquisition of new knowledge” (Desvallées and Mairesse, 2010, p. 31). It seems that the visiting experience can lead to long-term introspective and cognitive outcomes, especially in terms of social awareness (DeWitt and Storksdieck, 2008). During the museum experience, the visitors are involved in a process of discussion, interpretation, and negotiation of meaning in relation to the cultural heritage embedded in the place; they are part of an “interpretative community,” where meaning-making is mediated between individual and collective interpretations (Hooper-Greenhill, 2000). Paris (1997) suggests that social interaction facilitates visitor learning, enhances motivation and negotiation skills, and monitors accomplishment.

This premise is important to understand our research design and the interest we have in evaluating an aesthetic experience during a learning activity. The museum educational purpose affects all the activities that take place inside the museum and justifies the institutional choices of architecture and design. Moreover, these factors determine the interaction, cognitive understanding, and learning achieved in the transmission of museum contents. There are two reasons why our case study involves a sample of children: the first concerns the scarce literature of children visiting experience, although many of the museum’s educational and learning activities are dedicated to the schools’ target. The bond between museum and visitors is not taken for granted but is strictly related to the involvement with the community in which people live. This link should be encouraged and motivated emotionally during childhood. Bourdieu (1967), in his studies dedicated to the culture audience, tells us that we must create the “affection for cultural heritage” in children early in life because it is only when the presence of culture is registered in everyday life that it is missed (or sought) in adulthood. The educational department of MART is a national model in Italy. For this reason, we found it interesting to investigate the “environmental experience” during some learning activities dedicated to local primary schools.

The same lack of studies has been found regarding the issue of Restorativeness. The theory of Restorativeness has an aesthetic basis, which refers to the concept of fascination (Kaplan, 1987). Although we understand how Restorativeness affects adults, few studies have sought to describe the relationship between children and urban places or how this relationship could help to reexamine the cultural and learning environment. In our research, which focuses on the museum environment during children’s learning activities, we investigated the museum through its fascination attributes. In relation to learning settings, researchers highlight that providing children with access to environments that enhance and not merely support restorative processes, and which facilitate or optimize development and performance, is clearly beneficial to children (Bagot et al., 2015; Kelz et al., 2015).

### Reasons to Redefine the Aesthetic Episode During the “Museum Experience”

Through this research, we investigate the relationship between the aesthetic episode and the museum experience. In particular, we ask ourselves how and to what extent, during a museum visit, the environment participates in the success of the aesthetic episode. In fact, there is often a process of aesthetic evaluation of museum architecture and design in which the visitor is involved and at the same time not very aware. Often, the focus on museum educational dimension overshadows the context, yet the two are closely related (Mastandrea et al., 2019).

In the last 30 years, the concept of “education” in museums has been progressively expanded by museum professionals and academics to create a theoretical and methodological framework for interpreting learning activities in the museum environment (Allard and Boucher, 1998; Hein, 2002; Leinhardt et al., 2003). Dierking and Falk (1992, 2000) developed a “contextual model of learning”—the personal, sociocultural, and physical contexts, within the flow of time. Csikszentmihalyi and Robinson (1990) stressed the significance of the aesthetic experience, applying their “flow” model to museums. “Flow” is described as an authentic experience that occurs if people are deeply involved in a creative process. The original account of the state of flow has proved remarkably strong, confirmed in studies of art and aesthetic experience and many other recreational activities. Rather than focusing on the person, unrelated to context, “flow” research emphasizes the dynamic system of the person and the context.

Pekarik et al. (1999) studied the expectations that visitors bring to a museum and described the various elements that comprise the subsequent experience. Because of the fluidity and multidimensionality of the phenomenon, they developed the following four-part framework to encompass the concept of “museum experience:”

• Object experiences: in which the individual focuses on the content, the object, or “the real thing;”
• Cognitive experiences: in which the individual gains information or knowledge;
• Introspective experiences: in which the individual turns inward, to personal feelings, memories, and experiences, with a sense of belonging or connectedness;
• Social experiences: in which the individual interacts with family members, friends, and often museum staff.

Yet, context is extremely important. Combs (1999) suggested that learning and recreation are the primary reasons behind a museum visit. The experience of learning in a museum becomes one of discovery overlaid with personal and social elements that are also pleasant and enjoyable. Subsequently, visitor research has adopted this interactionist perspective and focuses not only on the activities carried out by visitors at the museum but also on the ways the museum environment in which the activities take place affect the visitors. This approach considers the observation that architecture and environmental design can affect people’s emotional states as well as the way they behave. Recent research with museum visitors has supported the notion that visiting art museums and exhibitions has an emotional impact on individuals exceeding beyond what is triggered by the objects on display. Observing extraordinary objects, moving in an unusual space, being surrounded by people—friends or strangers—who are similarly involved in interpreting what they see, these are all factors contributing to the pleasure of the experience.

We consider that the aesthetic process can also take place inside the “museum context” and because of it, despite the fact that classical theories focus primarily on evaluating the aesthetics of the object. The first psychologist to put forward an empirical approach to aesthetic appreciation was probably Fechner (1876), the creator of the “aesthetics from below” concept that focuses on the way in which an object’s perceived structural characteristics are appreciated by the observer. For Fechner, an object’s structure contains intrinsically aesthetic qualities such as proportion, symmetry, and complexity, which cause an individual to have a specific reaction and aesthetic preference (Tinio and Leder, 2009). In contrast, a subsequent “aesthetics from the top” model concerns an individual’s knowledge, expertise, emotional background, and personality traits, which also have a role in shaping the final experience (Mastandrea, 2014). In the 1970s, Berlyne introduced his psychobiological aesthetic theory based on the concept of “excitement” or arousal as a stimulus for curiosity and exploration. Object attributes such as originality, uncertainty, and ambiguity were considered legitimate elements in shaping the aesthetic experience (Berlyne, 1974). Recently, especially with the development of neuroaesthetics, a greater interest in the emotional component of an aesthetic experience has appeared in relevant literature. Leder et al. (2004), for example, suggested a descriptive model that describes how information is processed during an aesthetic experience on three levels: perceptive, cognitive, and emotional. The boundaries between cognitive and emotional experience become more subtle, and the aesthetic judgment is hardly distinguishable between subjective and objective opinions. Ten years later, Leder and Nadal (2014) reviewed the model highlighting the role of contextual factors on aesthetic experience. According to the authors this includes two aspects: the aesthetic judgment, based on cognitive process and correlated to the interpretation of the object (the artwork), and the aesthetic emotion, based on the emotional path experienced by the preceptor during the entire experience. The two could be confused, overlapped, or diverged in the preceptor’s mind.

The aesthetic episode also hides itself behind physiological sensations (Scherer, 2004): we can detect numerous examples of these reactions by observing behaviors and attitudes of visitors. Pekarik (2002) launched a reflection in the Curator Journal on the mental state involved in museum learning: “The mental state involved in emotionally responding to the object can be very different from the mental state involved in reading and thinking. While our desire to effectively facilitate meaning pushes us to emphasize communication through language, many museum experiences are firmly rooted in feelings that are not enhanced by words” (Pekarik, 2002, p. 263). Pekarik’s intention was to highlight the emotional response to a museum exhibit, suggesting that the visitor’s learning process could be much more about “feeling” than “thinking” or “explaining.” Hooper-Greenhill (2007) affirms that while learning in a museum, “mind and body work together;” it is clear that children experience the visit as “a physical experience, which engages their feelings and emotions and allows their minds to open up to new ideas” (p. 165). Roberts (1991, 1992) pays attention to visitors’ affective responses to their museum experience, such as sudden comments like “I really enjoyed it!,” “I had fun,” “It was boring,” “That visit really moved me.” Affective responses can also be demonstrated in visitors’ physical behavior such as the continuous or recurrent observation of an object. Some behaviors indicate an affective engagement by returning to look at an object, showing it to someone else, discussing its value, and comparing opinions with others. Presence and movement in the museum environment can be a clear indicator of the involvement of visitors.

It becomes increasingly difficult to categorize the sensations described by visitors, yet in these, we continually find important indicators of aesthetic experience to encourage future analysis. For example, the sense of inspiration, stature, and spirituality culturally attributed to aesthetic experience (Zeki, 1993, 2002) has a place in the museum experience. Some recent articles have shown that these experiences give the visitor a temporary sense of separation from reality and then a subsequent return to everyday life with renewed awareness: the sensation of being part of “something bigger” (Packer and Bond, 2010). In the light of these testimonies, the correlation between the aesthetic episode and the environment, according to the principles of Restorativeness, appears strong.

### Experiencing the Museum Environment: An Increasingly Important Aspect

Although the disciplines of Environmental Psychology and Visitor Studies have discussed the theme of the museum environment and the way in which it affects visitors at considerable length, we are still far from a recognized definition of what constitutes the “museum environment.” Among the 21 fundamental concepts of museology listed in the reference tool *Key Concepts of Museology* edited by ICOM’s International Committee for Museology (ICOFOM), we find the term “architecture” but not “environment.” “Architecture is defined as the art of designing and installing or building a space that will be used to house specific museum functions, more particularly the functions of exhibition and display, preventive and remedial active conservation, study, management, and receiving visitors. Since the invention of the modern museum, from the end of the 18th century and the beginning of the 19th, while old heritage buildings were also being reconverted for museum use, a specific architecture evolved that was linked to the requirements of preserving, researching and communicating collections through permanent or temporary exhibitions” (Desvallées and Mairesse, 2010, p. 24).

From a psychological perspective, it is useful to see the museum institution as an environment that “hosts” the visitor. Put more generally, any physical context becomes an essential part of the perceived experience, and every experience is a part of an individual’s interaction with their environment, both human and physical (Dewey, 1934). For this reason, the disciplines of Environmental Psychology and Visitors Studies are trying to expand the debate about the ways in which a visitor’s experience is moderated by the architecture and physical design of a museum. For example, Tröndle et al. (2012) showed that the experience of art in museums is closely related to the itinerary of visitors through space. Mastandrea et al. (2009) showed how the research environment (being in a laboratory rather than in a museum) changes the perception of art. Studies in which the museum environment is thought in terms of “customer experience” are more frequent. Doering (1999) discusses visitor needs in relation to a “setting” or “servicescape” that support and enhance visit experiences. “According to Bitner (1992), the servicescape, or service environment, includes ambient conditions such as temperature, lighting and noise; spatial layout and functionality; and signs and symbols such as the quality of furnishings which explicitly and implicitly convey expectations and ‘image.’ She suggests that these features influence customers’ (or visitors’) cognitive, emotional and physiological responses to the environment” (Packer, 2008, p. 34; Bitner, 1992).

We assume that the time has come to think of an “environmental experience,” in which the individual interacts with the museum spaces, moving around and enjoying the building architecture and the exhibition design in terms of aesthetic impact. Museum design is fundamental for a successful museum experience. A museum visit unfolds through movement in space: the environment determines how visitors explore, engage, contemplate, reflect, and understand exhibitions. The entire educational message depends on the perception of space. According to Nasar (1994), some of the architectural characteristics that are beneficial to the individual are the following:

• Visual quality: a space that is interesting, but not confusing, where its intriguing points are not immediately obvious but are revealed as people move through the space.
• Balance of order and complexity: individuals tend to like spaces that are neat and only moderately complex. A space is complex when there is variety in the spatial elements arranged without many color patterns.
• Naturalness: the implicit or explicit reference to nature, in the architectural structure, in the design choices, and also in the environmental conditions (such as natural light, the presence of water, adequate ventilation).

“The medium is the message” is a phrase coined by McLuhan and Fiore (1967), meaning that the form of a medium embeds itself in any message it wishes to transmit, creating a symbiotic relationship by which the medium influences how the message is perceived. Museums convey to visitors the message of cultural heritage and its values through cultural content (objects) and by facilitating certain cognitive, introspective, and social experiences. However, the museum experience is more than this; it incorporates the influences of the contextual physical environment. Thus, what is learned from exposure to a museum is a process of what the French literature describes as “Mediation Culturelle” (translated into English as Cultural Mediation or Interpretation). In the French literature, the term mediation is frequently used to refer to “a whole range of actions carried out in a museum context in order to build bridges between that which is exhibited (seeing) and the meanings that these objects and sites may carry (knowledge)” (Desvallées and Mairesse, 2010, p. 47).

With the new wave of contemporary museums and exhibition spaces developed by “starchitects” (the so-called “the Bilbao effect”), the relationship between the museum, the visitor, and the structure’s architecture and design can no longer be ignored (Rybczynski, 2002; Plaza, 2007). The architecture of the building and the design of the exhibition spaces mediates the messages from the objects contained in the museum (Sirefman, 1999; MacLeod, 2005). The museum architecture itself becomes a medium. “Post urban museum architecture cannot simply be a container; it must have content of its own. As a building in and of itself, the architecture need not compete with the art or artifacts on display; in fact, it can enhance the exhibition experience. These two needs container and architectural presence are not mutually exclusive; a museum can at once be a significant edifice and be sympathetic to its required functions” (Sirefman, 1999, p. 298). For this reason, we can say that the museum environment can be considered a medium itself (Ponzini, 2011, 2014). Increasingly, museums are consciously designed and built with the mediating role in mind.

### The Museum as a *Restorative* Experience Is Based on Fascination

In his discussion of attention, James (1892) observed that voluntary attendance to some stimuli took effort, an effort that we experience “…whenever we *resist the attractions* of more potent stimuli and keep our minds occupied with some object that is naturally unimpressive” (James, 1892, p. 224). This sense of effort has been understood throughout the ongoing study of attention to lead to fatigue. According to Attention Restoration Theory (ART) (Kaplan and Kaplan, 1989), for example, the need to continually focus attention produces mental exhaustion. This state, called “directed attention fatigue,” can give rise to irritability, anxiety, anger, frustration, inability to perform cognitive tasks, and increased errors in performance. Nevertheless, attention fatigue can be overcome in so-called “restorative environments” that evoke effortless attention (Berto et al., 2010). An important aspect of research findings linked to ART is that people often experience nature as being restorative. Being in natural settings (or even looking at images of natural settings) can lead to a reduction in mental fatigue. Natural environments arousing “fascination,” a condition in which a person can reflect in a state of *effortless attention*, abound. Fascination is the main attribute that an environment requires to be considered restorative, and it plays a crucial role in attention restoration theory (Kaplan, 1995). Fascinating stimuli are attractive, prevent boredom, and, most importantly, enable people to function without directing their attention (Berto, 2005).

Some studies have shown that museums have a high potential for fascination. Packer and Bond (2010) noted a significant overlap between museum attributes and those suggested by Kaplan (1995) as creating a restorative experience. The findings set out in their study indicate that for some people, museums can be as restorative as natural environments, thus providing insights into the factors that contribute to the visitor’s well-being. The phenomenon of restoration was further explored by questionnaires, collected after a visit, and from which the authors discovered that most visitors reported having attained a sense of relaxation and renewed ability to deal positively with life (Packer, 2008). It follows that a restorative condition can be extremely helpful for visitors involved in a museum learning process.

The relationships between the level of perceived restorativeness of an environment and its aesthetic evaluation have been documented in some studies. Galindo and Hidalgo (2005) revealed that “harmony,” “openness,” “brightness,” “suitability for leisure,” and “meeting place” correlated with perceived restorativeness. Much has been written about the selection of “favorite” places (Korpela and Hartig, 1996; Korpela et al., 2001) and on aesthetic judgments of places (Purcell et al., 2001; Peron et al., 2002). Hidalgo et al. (2006) identified categories for attractive and unattractive urban places. The research involved residents from two European cities who were asked to identify the most visually attractive and unattractive place in their city. The five main categories investigated were cultural–historical places/landscapes, recreational places for leisure and/or walking, places with a view, housing areas, and industrial places. Historic–cultural (48%) and recreational places (33%) were experienced as more aesthetic and restorative. These results have several implications. First, the study suggests that a museum environment could have the potential to be a restorative place due to its “historic–cultural” vocation and role. Second, the potential of a museum as an environment for learning might be enhanced because of the recreational aspect of a visit. Moreover, “Culture” and “Recreation” are two of the main categories of reasons given for visiting a museum in general. Using open-ended questions, Korpela (2002) asked children about their favorite places. The preference was for locations where activities and social interactions were available.

Kaplan et al. (1993) returned to past research and reanalyzed focus group comments about the museum experience, finding evidence of Restorative attributes:

• Fascination: places that require little or no attentional effort,
• Being away: taking a break from the daily routine,
• Extent (Scope and Coherence): a place that is rich and coherent enough to be explored,
• Compatibility: the extent to which an environment supports your inclinations and aims.

Based on an additional study, the researchers expanded the range of restorative outcomes to include feeling refreshed, restored, thoughtful, relaxed, and not feeling tired or worried. Packer and Bond (2010) analyzed the restorative attributes and benefits described by visitors in some important public learning institutions: art galleries, botanical gardens, parks, zoos, aquaria, and historic sites. On the basis of the study, the authors drew up a list of motivations that bring visitors to these cultural and recreational places: “learning and discovery,” “passive enjoyment,” “restoration,” “social interaction,” and “self-fulfillment.”

Contemporary research has begun to explore the role of restorative environments in the school setting (Bagot et al., 2015; Berto et al., 2015). If some aspects of current educational experience serve to train children in effortful directed attention, while others allow for the exploration that comes from involuntary attention, it is important to know which balance exists in museums. How might a visit to a museum be structured to maximize the cognitive, emotional, and social benefits to a child? The museum environment, through the attributes that trigger “fascination,” might induce in children a condition of effortless attention and consequently might also facilitate the learning process.

## A Pilot Study

This introduction has briefly outlined the progressive expansion of the museum communities’ understanding of the various psychological ways a museum might affect a visitor. One of the effects we have described at length is the way an environment can facilitate recovery from the fatigue caused by the effort of focusing attention on events or activities that do not of themselves attract attention. This case study, involving a small group of children engaged in a specially structured school visit at the MART, Museum of Modern and Contemporary Art of Trento and Rovereto, aims to investigate their perception of the museum as a restorative environment and its connection with children’s whole museum experience. In the context of discussing the education of children, we have indicated the restorative aspect of the school environment as attracting recent research attention. We have also put forward the general hypothesis that the architecture and design of a museum might provide restorative elements that affect other aspects of the aesthetic experience relevant to education.

### Promote the Architectural Heritage of MART: A Collaborative Museum Learning Project

The study is based on the evaluation of the museum visit as positive, profound, and enriching experience. We started from the assumption that a true museum experience cannot be reproduced in a laboratory. To increase the ecological validity of the founding, it was necessary to implement the study inside a museum as a real school learning program. DeWitt and Storksdieck (2008) demonstrated that cognitive and affective learning can occur as a result of class visits to out-of-school settings, such as the museum experience. They highlight that “learning outcomes are fundamentally influenced by the structure of the field trip, setting novelty, prior knowledge and interest of the students, the social context of the visit, teacher agendas, student experiences during the field trip, and the presence or absence and quality of preparation and follow-up” (p. 182). With MART Education Office approval, we created a special learning program called “EMOZIONI IN MOSTRA!,” which was included in their annual program for local schools. This inclusion undoubtedly contributed to promoting the initiative, encouraging enthusiasm and support from all participants: parents, teachers, and students.

Every learning school program includes educational objectives relating to acquiring skills, knowledge, aptitudes, etc. Some of the educational goals shared by our research group, the museum staff, and schoolteachers are inviting the children to take part in a museum experience inside MART and develop an idea of architectural heritage; explaining the role of the architect, the characteristics of architecture and interior design, encouraging the use of the senses to perceive the museum environment; improving children’s vocabulary to describe the MART architecture; improving children’s aesthetic judgment ability; and encouraging them to give their own graphic interpretation of the museum experience.

When designing the visit, we paid particular attention to the part dedicated to the learning activity (timing, modality, and contents) to facilitate the aesthetic experience. Following the analysis of Shusterman (1997), the case study was organized in order to highlight three main dimensions that confer an aesthetic quality to an experience: an evaluative dimension, that it involves the perception of an object (museum architecture); an affective dimension, about engagement, attraction, and attention (the visit and its learning activities); and a semantic dimension, in which an aesthetic experience became an interpretation via a meaning making process (the postvisit activities and the drawing realization) (Schorch, 2014).

All the activities were designed to allow children to give us their interpretation of the environmental museum experience. From the psychology of art to neuroaesthetics, the concept of interpretation has always been present in aesthetic debate. Arnheim (2002), and the Gestalt school before him, clearly states that artistic objects share with artists and users an interpretation of himself, suggested by the structure and the integrated shape qualities. The interpretation is one, and there is only one way to receive it correctly. Subsequently, Zeki (2004) recognizes the ambiguous interpretation: the fact that it is not necessary to achieve a “correct” or “unanimous” interpretation but recognizing the existence of many and evolving interpretations is a positive fact. Nowadays, interpretation is a recognized cognitive process that is part of the aesthetic experience and its search for meaning. Leder and Nadal (2014) assessed interpretation as an individual dimension while acknowledging the coexistence of a social dimension. In many aspects, art and aesthetics serve social functions, and museum environments itself can fulfill the need of social interpretation (Bourdieu, 1979).

During the educational program, children are accompanied in forming an interpretation of the museum experience individually, as a person, and collectively, as a class group. Within this interpretation, we look for the elements that refer to MART museum environment. “All material elements of an exhibition and the respective framings (building, specific location within a certain type of architecture, style of announcements) define the ways in which an exhibition becomes meaningful for the individual visitors, connecting the intended message with their specific repertoires of associations and connotations, and the pertinent and relevant social facts. Thus meaning and information for an exhibition visitor can only be produced within the complex and necessarily positive interaction of his/her own categories of thinking and experiencing and the forms offered in the exhibition (Umiker-Sebeok, 1994). The visitor will ‘see’ what is shown, and will see and interpret whatever is there within his/her own background of experiences and pre-knowledge. […] The idea that everything in a museum, all artifacts or object elements in the museum surrounding, exert sign functions is basic to an understanding of the museum as a semiotic communications system (Stránský, 1991)” (Weltzl-Fairchild, 1995, p. 66).

## Research Aims

During the research process, we focused exclusively on the architecture and design of the building, as an example of architectural construction, conceived and executed according to precise choices of style, meaning, purpose, and audiences. This aspect was considered interesting by the museum institution precisely because it had never been experienced before: this was an opportunity to test the MART’s Architectural Heritage potential. What are the factors that influence responses to the MART architecture and design? Galindo and Hidalgo (2005) distinguished attractive places from both aesthetic and restorative points of view. They showed that Nasar’s aesthetic attributes characterized the most attractive place in terms of openness, mystery, complexity, order, vegetation, maintenance, style, and perceived use. All the most restorative places presented these aesthetic criteria. Considering these results, we elaborated the following research aims. The first was to investigate the perceived restorativeness of a visit, by distinguishing among different physical environments inside the museum: the “Dome,” the “Bridge,” and the “Gallery.”

These three different environments were chosen in line with Nasar’s (1994) specification of the three attributes that a beneficial environment requires, namely, visual quality, the balance of order and complexity, and naturalness. Visual quality, which refers to a space that is interesting but not confusing, could be the most important attribute of the “Dome” to be considered in our case study. Considering the second attribute, that is the balance of order and complexity, it can be argued that the “Gallery” was the museum space that best represents this attribute, with white walls and natural light, which allow one to be immediately drawn to the artworks on display. Finally, naturalness, the imitation of natural elements, openness, and natural light, was an attribute present in all the three locations. The “Dome” has the sky and the fountain with running water; the “Bridge” recalls the shape of a tree and also contains a real tree; and the “Gallery” has large windows through which you can see the mountains and the forest and also has a wooden igloo.

These three attributes can also relate to restorativeness and its subdimensions. Visual quality, given its definition, can be considered to be connected with scope, fascination, and coherence. The balance of order and complexity recalls the dimension of coherence and, to some extent, fascination. Finally, naturalness is connected with being away and fascination.

These considerations lead to our research hypotheses: the museum architecture and design, given its physical characteristics, should show high level of restorativeness. Moreover, the different physical environments in the museum should differ in the level of the different subdimension of restorativeness. Specifically, the “Dome,” with higher level of visual quality, should show higher level of scope than the other two environments and a moderate level of fascination and coherence, and the “Gallery,” showing a good balance of order and complexity, should show higher level of coherence and fascination than the other two environments. All three environments, given the high level of naturalness, should be high in fascination and being away.

The second aim was to explore children’s “museum environmental experience,” more specifically, the way perceived restorativeness and preference reflected in the individual and collective interpretation/representations produced by the children.

The exploration of children’s “environmental preference” was conducted through different activities involving a direct self-measure of preference during the visit and a social activity called “the negotiated drawing” after the visit.

The exploration of children’s “environmental experience” was conducted through an individual activity called “the collage inside the head.” We classified their interpretation/representations of the whole museum experience, considering the four components, plus one: objective, introspective, cognitive, social, and environmental (Pekarik et al., 1999).

Regarding the environmental preference, we expected a correlation between the type of museum spot declared as “preferred” and the number of times this has been represented by children. Concerning the “environmental experience,” we assumed that the category has been represented on a par with the other four: objective, cognitive, introspective, and social.

## Materials and Methods

### Participants and Procedure

The research involved 41 children, 20 male and 21 female, from two primary school classes in Rovereto (Italy); mean age was 8.3 years old (SD xxx). The project comprised two phases: the visit (a tour in the museum) and the postvisit activity (a school activity after the museum visit). We want to specify that the group of children was a convenience sampling. We know that convenience sampling is not recommended for research due to the possibility of sampling errors and the lack of representation of the population. In these specific educational circumstances in partnership with the Mart, practical sampling was the only possible option.

### Stimuli: MART’s Design and Architectural Characteristics

**FIGURE 1:**
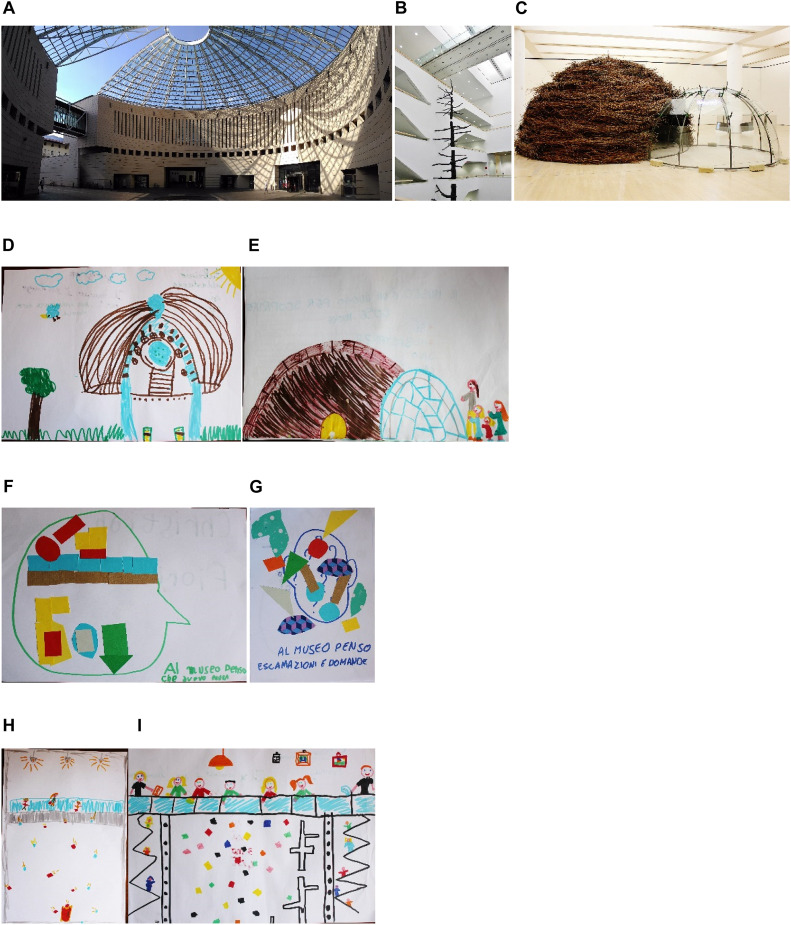
The three different physical environments inside the MART museum: the Dome **(A)**, the Bridge **(B)**, and the Gallery **(C)**. Two examples of negotiated drawing, representing the Dome **(D)** and the Gallery **(E)**. Two examples of collage. Panel **(F)** represents the Bridge: “When I think of the museum, I’m afraid to fall down.” Panel **(G)** represents some question marks and exclamation points: “When I’m at the museum, I think to exclamation and questions!” **(H,I)** Two examples of negotiated drawing, representing the Bridge during the visit learning activity.

MART was designed by the architect Mario Botta, in collaboration with the engineer Giulio Andreolli. The building is famous for its large glass and steel dome above the central access hall to the museum. Our research focused on three different environments considered representative of MART architecture in terms of its open spaces: “the Dome,” “The Bridge,” and “the Galley” (Figures 1A–C).

The environment and the visit contents were selected starting from two essays about the architecture of Mario Botta. The first book, an “unofficial” essay property of MART’s Educational Office, was chosen for its interviews with Mario Botta; the second because it was written by the architect himself (Botta and Andreolli, 1995). Both books include the direct words of the architect of MART and his creative approach. In them, he describes in detail the artistic vision and stylistic choices underlying the museum design.

Below, we describe the contents of the guided tour, extracted from the literature listed:

• *The Dome*. A long and narrow corridor connects the main road and MART main entrance. Mario Botta describes this corridor as an “umbilical cord” because the city of Rovereto encloses the museum as in a womb. In it the museum grows and evolves, disclosing its full potential. The architect uses this image to describe the close relationship between the museum, the city, and its citizens. Building such a long and narrow entrance, the architect wanted to play with contrast, surprising the visitor with the discovery of somewhere unexpected: the large circular square covered by a majestic dome of glass and steel. The dome has a hole at the center, from which it can filter sunlight and rain is filtered. It is difficult to know if you are outdoors or indoors. Mario Botta says that “we are a little inside, but also outdoors.” “The covered square may look like an outdoor space but is also a transition from the city and the museum.” Technically, we are still outside the museum but not quite in the town, ready to enter. “The square: upon entering from the street you immediately feel like you’re in a special place, prompting immediate reflections on its nature.” What is a museum? What is there inside? What can I do there? From the square, it is possible to access various museum spaces, with a series of doors all along the perimeter: “This beating heart is the hub through which all the various activities are functionally distributed: the museum, the library, the administration, the café, the reserved teaching spaces and the City auditorium.”
• *The Bridge.* This is the entrance to the museum: the real protagonist of the space is a staircase that ends with a glass bridge. “From the stairs, you can access the different levels (floors) through the side passageway or through the walkway on the top floor; the passageways give visitors an idea of the size of the vertical section of the building.” Via this walkway, the visitor crosses the museum at its highest point. Once on the bridge, the visitor can see the full breadth and height of the building, with the thrilling sense of a void. Mario Botta says: “The emptiness of the two juxtaposed vertical staircases make a vertical spine.” The staircases designed by Mario Botta allude symbolically to vegetable life, a solid, natural, branching structure.
• *The Gallery*. Mario Botta refers to the design choice for these spaces as “Exhibition hall nudity… where the architecture takes a step back to let Art and its protagonists talk.” The architect thinks of these spaces, generically bright and totally white as a stage: the works are actors playing their part in Art History. Nothing can distract the visitor from the observation of the artwork, not the wall, not the floor or the ceilings. “Inside the exhibition galleries, where the artworks are exhibited, appropriate lighting and neutral architecture prevent a babble of different languages and facilitate the direct contact between the art and the visitors.”

#### The Visit

The visit was scheduled for a weekday morning. In collaboration with the MART Educational Office, we chose a time when the museum was not crowded. In addition, MART’s Educational Office ensured no other tours were taking place in the same exhibition spaces during the visit. Thus, our activities were not interrupted or hampered in any way. The children were able to visit the museum in a peaceful and quiet atmosphere. The children moved in a group and were accompanied throughout the visit by an educator, so problems of wayfinding were mitigated.

The children’s museum tour was organized as follows to capture the children’s attention. At the beginning of the visit, each child received a small brochure with a selection of drawings and quotations from Mario Botta’s books. We asked them to imagine they were a group of judges, experts in architecture, invited to the museum to evaluate Mario Botta’s architecture and design. The children were guided in an architectural walk through three different museum locations: the Dome, the Bridge, and the Gallery. The total duration of the visit was 60 min. At each location, the activity was carried out in the same way: 7 min of architectural explanation, 3 min of “physical exercise,” 5 min to complete an assessment scale, and 5 min to get to the next place. The expression “physical exercise” means brief motor activity to engage the children: walking, running, throwing an object, and sitting down. It is not a novelty that aesthetic experience and aesthetic emotion are linked to movement. Chatterjee and Vartanian’s (2014) propose a model of “aesthetic triad” in which “the aesthetic experiences derive from the interaction between sensory-motor neuronal systems, evaluation of emotions and knowledge of meaning” (Leder and Nadal, 2014).

• *Activity 1 in the Dome*. After the architectural and design explanations, the children were invited to spread out around the fountain. On a signal, a hand clap from the educator, they began to walk in all directions, and then, with another clap of the hands, they changed direction. The class was then invited to sit around the fountain and to think about and discuss how they felt about moving in this space: walking in all directions, choosing a destination, changing it, and then gathering together at the center again.
• *Activity 2 on the Bridge.* After the architectural and design explanations, the children walked in pairs and stood along the sides of the bridge against the glass barriers. The pairs were divided into two, and the educator gave the children a small piece of paper, red on one side and white on the other. The children were invited simultaneously to throw it into the void and watch the pieces of paper falling to the ground below. Then, the class was asked to sit and think about how they felt suspended on the bridge and what they understood about the size of that space.
• *Activity 3 in the Gallery*. After the architectural and design explanations, the children were invited to observe Mario Merz’s artwork “Chiaro scuro” (1983) and think about the contrast of natural and artificial materials used by the artist. The children were invited to sit on the floor next to the igloo they liked best and were involved in a collective discussion: which of the two igloos seems more comfortable? Which of the two houses seems safer? In which of the two houses do I want to live? How do I feel about this artwork, in this room?

After each activity, the children were invited to reflect individually, without having to provide a response to the group (to avoid the possibility of influencing each other). At the end of every reflective moment, the children did the test.

#### The Postvisit Activity

The two postvisit activities in which the children were involved took place 2 weeks after the visit. The aim of the two activities was to help the children to formalize and express their interpretation of the “museum environmental experience.” Mixed techniques of data collection with children are not new to research on museum learning: “These visual and written statements provide a remarkable record of the pupils’ responses to the often wonderfully exciting things they have just experienced in the museum. Their work is spontaneous, fresh and immediate, capturing their joy and enthusiasm before these are overlaid by events” (Hooper-Greenhill, 2007, p. 185).

Denham et al. (2007) describes the main elements of children’s emotional competence as a gradual path: among this awareness of emotional experience, discernment of one’s own and others’ emotional states and emotional literacy are not obvious for the age of 8 years old. In terms of aesthetic judgment and in case of aesthetic emotions, it could be difficult for children to express themselves. We have chosen the drawing tool to analyze the process of evaluating the environmental and aesthetic experience, thus avoiding verbalization: “children’s drawings are used to access children’s opinions and experiences by focusing on their personal narrations and interpretations” (Einarsdottir et al., 2009, p. 217). We considered drawings as an effective means for children to explore and communicate their content understandings and “environmental preference:” “Focusing on drawing as meaning-making moves away from the discourse of drawing as representation and, instead, focuses on children’s intentions, considers the process of drawing, and recognizes children’s drawings as purposeful: ‘drawing thus becomes a constructive process of thinking in action, rather than a developing ability to make visual reference to objects in the world’ (Cox, 2005, p. 123)” (Einarsdottir et al., 2009, p. 218).

• During the first activity—the “negotiated drawing”—the children were asked to explain, describe, and discuss their point of view and listen to other points of view about the museum experience. For the first activity we chose, the “negotiated drawing” activity (Cox, 1994; Cox et al., 1995), it involves two participants drawing in pairs, on the same blank sheet. In this study, each child used three colors that could not be exchanged between them. In this way, children have to discuss what to draw and how to do the drawing. Specifically, they need to cooperate in completing the figures they wanted to draw, alternating in the use of the colors provided. Before drawing, each couple had to discuss and try to answer the following questions: “Whose places is this? What do I learn in this place? How do I feel in this place?,” the place being the museum after the museum experience. At the end of the discussion, each couple was asked to draw: “My visiting experience at the museum.”
• During the second activity—the “collage inside the head”—the children were asked to reflect about the same topic but individually. The children were involved in a book reading. Sitting in a circle, the children and the researchers read the book “What are you thinking about?” (Moreau, 2012) from the genre of “silent book,” that is books without text. Silent books are produced to encourage spontaneous narration by children. The storytelling becomes a collaborative process based on the graphic illustrations. Every page in “What are you thinking about?” is illustrated with a head, and the reader sees the thoughts inside the character’s head. After the book reading that introduced the children to the concept of visualizing thoughts, the children drew the shape of a face on a sheet of paper then, using small pieces of paper of various shapes and colors, made a collage to compose some “thoughts” inside the outline of the head. Researchers used the prompt “When I think of the museum…” and asked them to complete the sentence with their clearest memories of the museum experience.

### Measures and Coding

Three different measures were used to analyze each aspect of the research: the first was a quantitative tool, whereas the other two were qualitative. The latter required a coding system designed specifically for research applied to the educational context:

• To understand the restorative attributes perceived by the children during the visit, we used the Perceived Restorativeness Scale—Children (PRS-ch), designed for school children (Berto et al., 2015). The scale was inspired by the Kaplans’ theories, based on the ART and the adult version consisting of 17 items describing four restorative factors: being away, fascination, coherence, and scope. A 4-point Likert scale was used (from 0 to 4, where 0 = “completely disagree” and 4 = “completely agree” (Pasini et al., 2014). The children were asked to think about how true each statement was for them and to tick the number corresponding to their judgment. The PRS-ch scale was their way of judging: they gave each sentence (item) a 1 to 4 rating, depending on how much they agreed with the statement about each specific environment, from 0 to 4, where 0 = “completely disagree” and 4 = “completely agree.” Preference was assessed as well, considered by us as an insight to the aesthetic preference, using a single item (“I like this place”) and a 4-point Likert scale. We called these data the “environmental preference.”
• The appreciation of museum architecture and design was evaluated back at school after the visit, using the “negotiated drawings” produced by the children. We have considered the representation of one space rather than another as a choice. The drawings were also used as an indicator of the most relevant memory of the museum environment and compared with the results of the “environmental preference.”
• To understand the interpretation of “environmental experience” in the museum, we used the collages. These were classified by researchers into five categories taken from the Museum Experience definition by Pekarik et al. (1999): (1) objective: explicit reference to museum artworks; (2) cognitive: explicit reference to exhibit information; (3) introspective: explicit reference to personal memories, emotions, and reflections; (4) social: explicit reference to classmates, teachers, museum educators, or visitors; and (5) environmental: explicit reference to museum spaces, design, and architecture. By “explicit reference to” we mean clear graphic elements (present in the drawing) and written words (in the title given to the collage). Three independent researchers analyzed the definition of museum experience (Pekarik et al., 1999) and the component categories, identifying the elements in each. They then looked for these elements in the drawings. In the event of judges’ disagreement, they discussed the collages until agreement was reached.

The enormous challenge of classifying museum experience was immediately clear to us. There is no one museum experience, pure and simple; it is a multidimensional phenomenon. All the elements characterizing personal visiting experience are closely linked.

### Data Analysis

We conducted a repeated measures ANOVA to test whether the perceived restorativeness (total score and component factors) and preference differed in the three environments. To locate the sources of the global differences reflected by the repeated measures ANOVAs, we evaluated the differences between the environments with a series of paired *t*-tests. In cases of statistically significant differences, we computed partial eta squared ($\eta_{\text{p}}^{2}$) for repeated measures ANOVA and Cohen’s *d* for *post hoc* analysis (Cohen, 1988; Tabachnick and Fidell, 2013). In agreement with Cohen’s criteria (Cohen, 1988), effect sizes were evaluated as negligible ($\eta_{\text{p}}^{2}$ < 0.01; *d* < 0.20), small (0.01 ≤ $\eta_{\text{p}}^{2}$ < 0.06; 0.20 ≤ *d* < 0.50), medium (0.06 ≤ $\eta_{\text{p}}^{2}$ < 0.14, 0.50 ≤ *d* < 0.80), or large ($\eta_{\text{p}}^{2}$ ≥ 0.14, *d* ≥ 0.80).

To evaluate museum experience, we considered children’s drawings, looking at the environment they decided to represent, and museum experience categorization, using descriptive statistics.

## Results

### Perceived Restorativeness

Our primary interest was to explore the level of restorativeness perceived by the children during the museum visit. The perceived restorativeness level was quite high for two of the three environments, the Dome and the Gallery, while the third environment, the Bridge, had a slightly lower level (Table 1).

**TABLE 1:** Mean level of the Perceived Restorativeness Scale (PRS) in the three museum environments (*N* = 41).

**PRS level**		
	**Mean**	**SD**
Dome	3.03	0.4
Bridge	2.63	0.8
Gallery	2.95	0.8

To test whether the perceived restorativeness differed in the three environments, a repeated measure ANOVA was run, considering the three different spots as the within subject factor. The results showed a significant effect of the environment [*F*(2,78) = 11.053, *p* < 0.001]. The effect size was large: $\eta_{\text{p}}^{2}$ = 0.22. The *post hoc* analysis highlighted that the Bridge was the least restorative environment, and the Dome and Gallery were equally restorative (*p*_B_ < 0.001). Effect size was from medium to large: Cohen’s *d* was 0.694 for the difference between the bridge and the dome and 0.827 for the difference between the bridge and the gallery.

**FIGURE 2:**
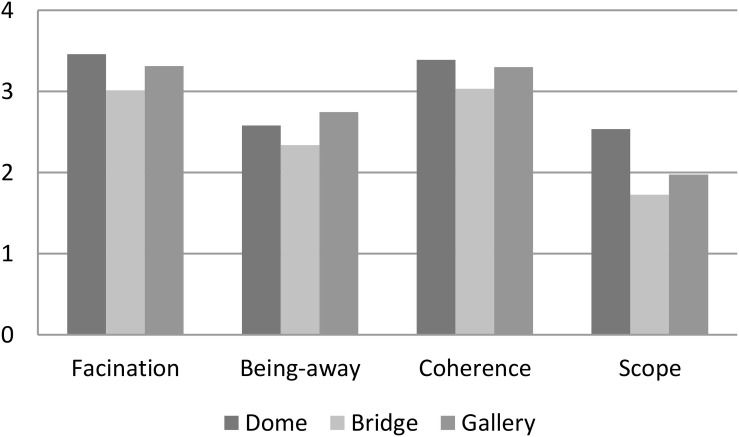
Mean level of the four restorative factors in the three museum environments (*N* = 41).

In a second step, we decided to consider the four restorative factors separately for each spot in order to understand which was the most and which the least restorative factor in the children’s museum experience. Fascination and coherence seem to be the two most prominent factors; scope was the least prominent factor for all the three environments (Table 2 and Figure 2).

**TABLE 2:** Mean level of each restorative factor in the three museum environments (*N* = 41).

	**Fascination**	**Being-away**	**Coherence**	**Scope**
Dome	3.46	2.58	3.39	2.54
Bridge	3.01	2.34	3.03	1.73
Gallery	3.31	2.75	3.30	1.98

To test whether these differences are statistically significant, a repeated measure ANOVA 3 × 4 was run, with two within factors: the “environment” (with three levels: Dome, Bridge, and Gallery) and the “restorative factor” (with four PRS’s dimensions: being away, fascination, coherence, and scope). The main effect of environment was significant: *F*(2,78) = 12.23, *p* < 0.001, with a large effect size ($\eta_{\text{p}}^{2}$ = 0.24). This result depends on the fact that the Bridge was the less restorative environment (see the previous result).

The main effect of “restorative factor” [*F*(3,117) = 48.00, *p* < 0.001], with a large effect size ($\eta_{\text{p}}^{2}$ = 0.55), is due to the lower level of scope (2.1), followed by being away (2.6), coherence (3.2), and fascination (3.3).

In addition, the interaction “environment” × “restorative factor” was significant, albeit with a small effect size: *F*(6,234) = 3.69, *p* < 0.01, $\eta_{\text{p}}^{2}$ = 0.09. Descriptive statistics showed that the Dome had the highest level of restorativeness for all restorative factors except for being away, where the Gallery had a higher evaluation. As shown in Figure 2, the PRS measures restorativeness in descending order: for the Dome, followed by the Gallery, and finally the Bridge. This is true for every PRS factor, except for being away (B-A), which is slightly higher in the Gallery. An interaction effect is produced by the Scope that seems to be much higher in the Dome than in the other two spaces.

### The Museum Experience: Preference and Categorization

In addition, preference showed difference between the three environments [*F*(2,78) = 4.10, *p* = 0.020, $\eta_{\text{p}}^{2}$ = 0.10] with the Bridge that obtained a lower score than the Dome [*t*(40) = 2.20, *p* = 0.034, *d* = 0.34] and the Gallery [*t*(39) = 2.69, *p* = 0.010, *d* = 0.40]. Table 3 shows these results.

**TABLE 3:** Mean level of environmental preference in the three museum environments.

**Environmental preference**		
	**Mean**	**SD**
Dome	3.65	0.6
Bridge	3.25	1.1
Gallery	3.58	0.9

The drawings were also classified according to the environment represented by the children: Table 4 shows the number of drawing for each environment.

**TABLE 4:** Number of drawings for each environment.

**Subject**	**Number of drawings**
Dome	9
Bridge	10
Gallery	12
Museum	10

The choice of which environment they decided to draw was compared with the preference levels. The results show that children drew the three environments equally, and their choice was evenly distributed over the Dome, Bridge, Gallery, and Museum (the category added for children who represented the museum as a generic place: a building viewed from the outside).

Although the Dome was the least chosen subject, it was experienced as the favorite spot standing on the preference response.

Finally, collages’ categorization is shown in Table 5. This table shows the distribution of each children’s collage, separately for boys and girls.

**TABLE 5:** Museum experience categorization.

Introspective	22	13M/9F
Cognitive	6	4M/2F
Objective	9	1M/8F
Environmental	3	1M/2F
Social	0	0M/0F
Other	1	1M/0F

After categorizing the collages, we created a new category, “Others’ experiences,” to classify the collage of a child who did not represent anything with implicit or explicit references to the museum visit (a child with behavioral problems produced this collage).

## Discussion

The findings indicate that the MART environment possesses aesthetic and restorative attributes. The test results showed that there were elements of the environment that triggered “fascination” in all three environments considered during the visit. The results, however, have not totally confirmed our hypotheses about the three kinds of Nasar’s formal aesthetic variables: visual quality, balance of order, and naturalness. This is probably due to the fact that these concepts are very fluid in the MART space.

In particular, the Dome had the highest overall score for three of the four restorativeness factors: fascination, coherence, and scope. This outcome may be due to the design characteristics of the Dome environment: a bright open space that invites exploration. On the other hand, the Bridge appeared to be the least restorative location with the lowest levels for all four restorative factors, probably due to the constraints of the physical space. It is narrow, high above a void, where movement is impossible and is devoid of elements with which one can interact. The highest of the PRS scores for the Bridge was for coherence, which was slightly higher than the score for Fascination. This result is probably due to the statement number 15 in the scale. That is, “in this place, it is easy to see what’s around me.” Due to its elevated and central position, the Bridge provides an open view of the various exhibition galleries. Scope was the least prominent attribute (equal to being away for the Dome). Scope was assessed by two PRS items: number 6—“In this place I am free to play, run and move,” and number 8—“This place is big enough to be explored.” This result was not surprising: running and playing are not the kind of actions open to visitors in a museum. The Dome, where children perceived a higher level of freedom to move around the square, was an exception.

We expected that the “Gallery” would show a good balance of order and complexity and, consequently, a higher level of coherence and fascination than the other two environments. Surprisingly, the Gallery scored highest in terms of being away. This result may be due to the fact that the gallery was the only space where the learning activity included the observation of an object—the artwork. Because of this, in the exhibition galleries, children were much more focused on something beyond themselves. The other two environments engaged children with a learning activity much more associated with the perception of their own presence in the museum space.

Turning to the postvisit activities, we found that “the negotiated drawings” represented a concrete, complex, and dynamic visit experience. All of them included elements of visual quality, balance of order and complexity, and naturalness. In particular, the children measured themselves through the representation of the museum environment (and the activity that took place in it) that had most affected them. We could clearly recognize the Dome, the Bridge, and the Gallery for their “formal aesthetic” attributes (Figures 1D–G).

Comparing the environmental preferences expressed by the children, we saw that the Dome was the most appreciated environment. This result is not surprising given that the Dome was also perceived as the most restorative place. Despite this, the relatively high number of drawings representing the Bridge indicated that the Bridge had a marked an impact on the children. However, as seen from the PRS scores, it was not a restorative element that seemed to cause the impact.

Obviously, a place can be appreciated without being restorative by virtue of other characteristics. To explore this issue, we analyzed the negotiated drawings in order to understand what kinds of feelings children experienced during the Bridge activity. Although the bridge was scary and gave some children a sense of vertigo, the activity was great fun for the children. The drawings show details of all the elements involved in the Bridge experience: the structure, the staircase, and classmates (Figures 1H,I).

The feelings were clearly impressed in their memory and consequently came out in their drawings. Therefore, it can be assumed that the Bridge experience provoked strong emotional and contrasting feelings, not of a restorative nature but nonetheless positive. Russell (2003) suggests that the key to understanding people’s response to the environment is through emotion. He describes the concept of “environmental affect,” comprising two main components: pleasure and arousal, subsequently described as “core affect” (Russell, 2003). Affective reactions to a place can be described by a model of emotion (Russell, 1988) based on contrast: pleasant/unpleasant and stimulating/sleepy. Affective qualities, described by an adjective, are based on a combination of these two dimensions: “exciting” is the combination of stimulation and pleasure (Roe, 2008). An additional element is whether or not one feels a sense of control of what might happen in an environment. Mehrabian and Russell (1974) included this element in their Pleasure, Arousal, and Dominance (PAD) model. The introduction of the “dominance” dimension reflects the extent to which a person feels in control or at risk in a particular environment. It is likely that one aspect of the Bridge experience included a sense of the possible lack of control.

Indeed, the Bridge is not a restorative environment; it was not comfortable or relaxing, but it was exciting (Berlyne, 1974). On the basis of appraisal theories of emotion, some authors suggest that negative emotions can bring of aesthetic feelings (Silvia, 2009, 2010, 2012).

Regarding the collage, despite the fact that we involved children in a specific test activity about MART architecture, Environmental only attracted three children’s collage. Among the Environmental collages, only one mentioned a specific museum spot: “I think of the fountain in the square,” referring to a large fountain in the middle of the MART area under the Dome.

We chose Objective experience for the collages that represented an artwork and mentioned it in the title: nine children, one boy and eight girls. Interestingly, this is the result with the largest gender gap between boys and girls. Of these nine children, six mentioned the artwork by name: “In the museum I think of the Rotating Head,” “In the museum I think of…the Strength of the curve.” The other three described and represented the object clearly, so they were evidently impressed by the object, even without the specific name. In the category Cognitive experience, we placed the collages that mentioned the learning process as the most satisfactory experience: to gain new information, acquire notions of art, expand personal knowledge, and reflect on inputs. Some children felt particularly engaged in the learning process, producing enthusiastic titles: “In the museum, I think about exclamations and questions!,” “In the museum I learn to learn,” “I learn to be happy in the museum!”). All collages that specifically named emotions during the visit were placed in the Introspective category: “I feel very happy in the museum! I think of all the colors and fun stuff!,” “In the museum I’m bored…,” “I think in the museum that I was afraid.”

During the collage activity, most children produced representations of an introspective visit (21 collages). The environmental element disappeared almost completely (three collages), and the social element was not represented at all. This type of result suggests that, given a very similar prompt (“My experience at the museum”/“When I think of the museum…”) but changing the method of expressing recollections of experience (individual or social), the museum representation changes.

## Conclusion

Our argument in this paper has been that as the understanding of the psychological relationships between people and museums has evolved, the effects of the physical “envelope that holds the objects” have come into focus more clearly. Not only the buildings but also the surrounding landscape has been shown to shape our experience of visiting a museum.

Through this museum learning experience—involving children and their parents, teachers, and a museum learning team, researchers were also able to explore some unanswered research questions in museum studies. We followed a psychological approach, applying the theoretical framework of Educational and Environmental Psychology and exploring some topics for the first time in a museum context. Field observations in an ecological research environment were analyzed combining the quantitative and qualitative perspective.

Our aim included developing an understanding of how the museum environment, when consciously exploited, can have a positive impact on children and their learning process. Our empirical work has begun to explore the links between museum design and restorativeness experienced by visitors. MART as a museum can be consider a “restorative environment,” an escape, a refuge, a break from the routine of daily life (Kaplan, 1995; Packer and Bond, 2010). Through a high level of fascination, the museum gave the opportunity to children to perform learning tasks in a condition of effortless attention. The result was remarkably interesting because it supports the restorativeness theory of learning environments designed for children (Berto et al., 2015).

Finally, our research confirms the relationship between restorativeness and museums (Packer and Bond, 2010), noting a significant overlap between museum attributes and those suggested by Kaplan (1995) for a restorative experience. The findings indicate that for most children, museums provide insights into the factors that contribute to well-being. Our research supports the idea of museums as places that contribute to a visitor’s aesthetic experience with a sense of relaxation, peace and calm, or thoughtfulness, but not only that.

Of the three environmental settings investigated, the Dome was the place with most restorative attributes. Classified as an outdoor environment, its natural characteristics may have contributed to this success. However, the Gallery also provided opportunities for regenerative experience, too, with the highest score for being away. Presumably, the presence of artworks helped children to live the sensation of escape and refuge, away from the routine of daily life. Focusing in particular on the restorative benefits perceived by children during the visit, we were surprised by the result for the Bridge, experienced by children as both terrifying and exciting. Due to this unexpected result, we reassessed the museum as a place able, among other things, to provoke strong aesthetic emotions and arousal. Further qualitative investigations can be made on drawings and collages made by children: categorizing the elements represented within can help to deepen the link between aesthetics and environmental preference (Lackney, 2000). Although our research asked children to reflect by drawing on the museum environment during the visit, we discovered that this Environmental experience was not particularly significant in young visitors’ memories.

The research has some limitations. It was challenging to reconcile different disciplines: the Philosophy of Culture, Museum Education, Environmental Psychology, and Educational Psychology. The need to adopt an interdisciplinary approach was dictated by the wish to take Museum Studies further by integrating quantitative theory and methods from psychology.

Moreover, mainly due to the lack of time and financial resources, the research was carried out quickly as pilot project. It would be interesting to extend this research topic in the future with a larger sample of visitors and over a longer period of time, including a control group. Although the research staff gave children their full attention and care, many children needed a more relaxed atmosphere and more time to visit the museum and its exhibits. A “control group” would have been very useful, but it was not possible to include the activity for local schools, which had already scheduled extracurricular activities for the current year.

Finally, the study was carried out in one museum alone, and it is certainly possible that the satisfying experiences and restorative elements identified in this particular museum are lacking in others. These results are valid and restricted to the MART—Museo d’arte moderna e contemporanea di Trento e Rovereto. Such a small study cannot make too many generalizations and is essentially preliminary research opening up new paths of enquiry for detailed examination. From the perspective of ecological validity, it would be desirable to use a sample of museums and be able to generalize findings across the wide spectrum of institutions that are categorized as being a “museum.” Nevertheless, the findings should encourage further research into the important and beneficial psychological effects that can be derived from visiting a museum.

## Data Availability Statement

The datasets generated for this study are available on request to the corresponding author.

## Ethics Statement

The studies involving human participants were reviewed and approved by Ethical Board of the Department of Human Sciences, University of Verona. Written informed consent to participate in this study was provided by the participants’ legal guardian/next of kin.

## Author Contributions

CA drafted the manuscript and wrote the sections of the literature review, the case study, and results. EM analyzed the data set. RH contributed in writing the theoretical framing, the discussion, and to the manuscript refinement. MP contributed in conceiving the rationale of the article as a whole, especially the Materials and Methods, and was involved in all sections of the manuscript. All authors contributed to the article and approved the submitted version.

## Conflict of Interest

RH was employed by the company Environmetrics Pty. Ltd., Australia. The remaining authors declare that the research was conducted in the absence of any commercial or financial relationships that could be construed as a potential conflict of interest.

## References

[B1] Allard M. Boucher S. (1998). *Éduquer au musée: un modèle théorique de pédagogie muséale*, Canada: HMH (Vol. 119).

[B2] Arnheim R. (2002). *Arte e percezione visiva. Nuova versione*, Milan: Giangiacomo Feltrinelli (Vol. 23).

[B3] Bagot K. L. Allen F. C. L. Toukhsati S. (2015). Perceived restorativeness of children’s school playground environments: Nature, playground features and play period experiences. *J. Environ. Psychol.* 41 1–9. 10.1016/j.jenvp.2014.11.005

[B4] Berlyne D. E. (1974). *Studies in the new experimental aesthetics: Steps toward an objective psycholoy of aesthetic appreciation.* Washington, DC: Hemisphere Publishing Corporation.

[B5] Berto R. (2005). Exposure to restorative environments helps restore attentional capacity. *J. Environ. Psychol.* 25 249–259. 10.1016/j.jenvp.2005.07.001

[B6] Berto R. Baroni M. R. Zainaghi A. Bettella S. (2010). An exploratory study of the effect of high and low fascination environments on attentional fatigue. *J. Environ. Psychol.* 30 494–500. 10.1016/j.jenvp.2009.12.002

[B7] Berto R. Pasini M. Barbiero G. (2015). How does Psychological Restoration Work in Children? An Exploratory Study. *J. Child Adolesc. Behav.* 3:200.

[B8] Bitner M. J. (1992). Servicescapes: The impact of physical surroundings on customers and employees. *J. Market.* 56 57–57. 10.2307/1252042

[B9] Botta M. Andreolli G. (1995). *Il Museo di arte moderna e contemporanea di Trento e Rovereto.* Switzerland: Skira.

[B10] Bourdieu P. (1967). *Systèmes d’enseignement et systèmes de pensée. Ecole pratique des hautes études.* New Delhi: Centre de sociologie européene.

[B11] Bourdieu P. (1979). *La Distinction. Critique Sociale du Jugement*. Paris: Les Editions de Minuit.

[B12] Chatterjee A. Vartanian O. (2014). Neuroaesthetics. *Trends Cogn. Sci.* 18 370–375. 24768244 10.1016/j.tics.2014.03.003

[B13] Cohen J. (1988). *Statistical power analysis for the behavioral sciences*, 2nd Edn Hillsdale, NJ: Erlbaum.

[B14] Combs A. A. (1999). Why do they come? Listening to visitors at a decorative arts museum. *Museum J.* 42 186–197. 10.1111/j.2151-6952.1999.tb01140.x

[B15] Cox M. Cooke G. Griffin D. (1995). Teaching children to draw in the infants school. *J. Art Des. Educat.* 14 153–163. 10.1111/j.1476-8070.1995.tb00621.x

[B16] Cox M. V. (1994). The Teaching of Drawing in the Infants School: An Evaluation of the “Negotiated Drawing” Approach. *Int. J. Early Years Educat.* 2 68–83. 10.1080/0966976940020104b

[B17] Cox S. (2005). Intention and meaning in young children’s drawing. *Int. J. Art Des. Educat.* 24 115–125. 10.1111/j.1476-8070.2005.00432.x

[B18] Csikszentmihalyi M. Robinson R. E. (1990). *The art of seeing: An interpretation of the aesthetic encounter.* California: Getty Publications.

[B19] Denham S. A. Bassett H. H. Wyatt T. (2007). “The Socialization of Emotional Competence,” in *Handbook of socialization: Theory and research*, eds Grusec J. E. Hastings P. D. (New York: The Guilford Press), 614–637.

[B20] Desvallées A. Mairesse F. (2010). *Key Concepts of Museology*, Paris: ICOM.

[B21] Dewey J. (1934). “Art as Experience, reprinted in 1989,” in *The Later Works, 1925–1953.* ed Edn, Boydston J. (Carbondale: Southern Illinois University Press).

[B22] DeWitt J. Storksdieck M. (2008). A short review of school field trips: Key findings from the past and implications for the future. *Visit. Stud.* 11 181–197. 10.1080/10645570802355562

[B23] Dierking L. D. Falk J. (2000). *Learning from museums: Visitor experiences and the making of meaning.* Walnut Creek, CA: AltaMira Press.

[B24] Dierking L. D. Falk J. H. (1992). Redefining the museum experience: the interactive experience model. *Visit. Stud.* 4 173–176.

[B25] Doering Z. D. (1999). Strangers, guests, or clients? Visitor experiences in museums. *Museum J.* 42 74–87. 10.1111/j.2151-6952.1999.tb01132.x

[B26] Einarsdottir J. Dockett S. Perry B. (2009). Making meaning: Children’s perspectives expressed through drawings. *Early Child Develop. Care* 179 217–232. 10.1080/03004430802666999

[B27] Fechner G. T. (1876). *Vorschule der Ästhetik.* Leipzig: Breitkopf und Härtel.

[B28] Galindo M. P. Hidalgo M. C. (2005). Aesthetic preferences and the attribution of meaning: Environmental categorization processes in the evaluation of urban scenes. *Int. J. Psychol.* 40 19–26. 10.1080/00207590444000104

[B29] Hein G. E. (2002). *Learning in the Museum.* United Kingdom: Routledge.

[B30] Hidalgo M. C. Berto R. Galindo M. P. Getrevi A. (2006). Identifying attractive and unattractive urban places: categories, restorativeness and aesthetic attributes. *Medio Ambiente Comportamiento Humano* 7 115–133.

[B31] Hooper-Greenhill E. (2000). Changing values in the art museum: Rethinking communication and learning. *Int. J. Herit. Stud.* 6 9–31. 10.1080/135272500363715

[B32] Hooper-Greenhill E. (2007). *Museums and education: Purpose, pedagogy, performance.* United Kingdom: Routledge.

[B33] James W. (1892). *Text Book of Psychology.* London: Macmillan & Co. Ltd.

[B34] Kaplan R. Kaplan S. Brown T. (1989). Environmental preference: A comparison of four domains of predictors. *Environ. Behav.* 21 509–530. 10.1177/0013916589215001

[B35] Kaplan S. (1987). Aesthetics, affect, and cognition: Environmental preference from an evolutionary perspective. *Environ. Behav.* 19 3–32. 10.1177/0013916587191001

[B36] Kaplan S. (1995). The restorative benefits of nature: Toward an integrative framework. *J. Environ. Psychol.* 15 169–182. 10.1016/0272-4944(95)90001-2

[B37] Kaplan S. Bardwell L. V. Slakter D. B. (1993). The museum as a restorative environment. *Environ. Behav.* 25 725–742. 10.1177/0013916593256004

[B38] Kaplan S. Kaplan R. (1989). The visual environment: Public participation in design and planning. *J. Soc. Issues* 45 59–86. 10.1111/j.1540-4560.1989.tb01533.x

[B39] Kelz C. Evans G. W. Röderer K. (2015). The restorative effects of redesigning the schoolyard: A multi-methodological, quasi-experimental study in rural Austrian middle schools. *Environ. Behav.* 47 119–139. 10.1177/0013916513510528

[B40] Korpela K. (2002). *Handbook of environmental psychology*, New Delhi: Wiley, 363–373.

[B41] Korpela K. Hartig T. (1996). Restorative qualities of favorite places. *J. Environ. Psychol.* 16 221–233. 10.1006/jevp.1996.0018

[B42] Korpela K. M. Hartig T. Kaiser F. G. Fuhrer U. (2001). Restorative experience and self-regulation in favorite places. *Environ. Behav.* 33 572–589. 10.1177/00139160121973133

[B43] Lackney J. A. (2000). “Learning Environments in Children’s Museums: Aesthetics, Environmental Preference and Creativity,” in *in Paper presented at “Beauty, Creativity and Sensory Delights* (Maryland: Institute for Civil Society).

[B44] Leder H. Belke B. Oeberst A. Augustin D. (2004). A model of aesthetic appreciation and aesthetic judgments. *Br. J. Psychol.* 95 489–508. 10.1348/0007126042369811 15527534

[B45] Leder H. Nadal M. (2014). Ten years of a model of aesthetic appreciation and aesthetic judgments: The aesthetic episode-Developments and challenges in empirical aesthetics. *Br. J. Psychol.* 105 443–464. 10.1111/bjop.12084 25280118

[B46] Leinhardt G. Crowley K. Knutson K. (2003). *Learning conversations in museums*, New Jersey: Lawrence Erlbaum Associates.

[B47] Linnenbrink-Garcia L. Pekrun R. (2011). Students’ emotions and academic engagement: Introduction to the special issue. *Contemp. Educat. Psychol.* 36 1–3. 10.1016/j.cedpsych.2010.11.004

[B48] MacLeod S. (2005). *Rethinking museum architecture: towards a site-specific history of production and use. In Reshaping Museum Space.* United Kingdom: Routledge, 23–39.

[B49] Mastandrea S. (2014). “How emotions shape aesthetic experiences,” in *The Cambridge Handbook of the Psychology of Aesthetics and the Arts*, eds Tinio P. Smith J. (Cambridge: Cambridge University Press), 500–518. 10.1017/cbo9781139207058.024

[B50] Mastandrea S. Bartoli G. Bove G. (2009). Preferences for ancient and modern art museums: Visitor experiences and personality characteristics. *Psychol. Aesthet., Creat. Arts* 3 164–173. 10.1037/a0013142

[B51] Mastandrea S. Fagioli S. Biasi V. (2019). Art and psychological well-being: linking the brain to the aesthetic emotion. *Front. Psychol.* 10:739. 10.3389/fpsyg.2019.00739 31019480 PMC6458291

[B52] McLuhan M. Fiore Q. (1967). The medium is the message. *N Y* 123 126–128.

[B53] Mehrabian A. Russell J. A. (1974). *An approach to environmental psychology.* Cambridge: the MIT Press.

[B54] Moreau L. (2012). *“A che pensi?”, libri con le finestre.* Bologna: Orecchio Acerbo.

[B55] Nasar J. L. (1994). Urban design aesthetics: The evaluative qualities of building exteriors. *Environ. Behav.* 26 377–401. 10.1177/001391659402600305

[B56] Packer J. (2008). Beyond learning: Exploring visitors’ perceptions of the value and benefits of museum experiences. *Curator Museum J.* 51 33–54. 10.1111/j.2151-6952.2008.tb00293.x

[B57] Packer J. Bond N. (2010). Museums as restorative environments. *Curator Museum J.* 53 421–436. 10.1111/j.2151-6952.2010.00044.x

[B58] Paris S. G. (1997). Situated motivation and informal learning. *J. Museum Educat.* 22 22–27. 10.1080/10598650.1997.11510356

[B59] Pasini M. Berto R. Brondino M. Hall R. Ortner C. (2014). How to measure the restorative quality of environments: The PRS-11. *Proce Soc. Behav. Sci.* 159 293–297. 10.1016/j.sbspro.2014.12.375

[B60] Pekarik A. J. (2002). Feeling or learning? *Curator Museum J.* 45 262–264. 10.1111/j.2151-6952.2002.tb00063.x

[B61] Pekarik A. J. Doering Z. D. Karns D. A. (1999). Exploring satisfying experiences in museums. *Curator Museum J.* 42 152–173. 10.1111/j.2151-6952.1999.tb01137.x

[B62] Peron E. Berto R. Purcell T. (2002). Restorativeness, preference and the perceived naturalness of places. *Med. Amb. Comport. Hum.* 3 19–34.

[B63] Plaza B. (2007). *The Bilbao effect.* Spain: Guggenheim Museum Bilbao.

[B64] Ponzini D. (2011). Large scale development projects and star architecture in the absence of democratic politics: The case of Abu Dhabi. *UAE. Cities* 28 251–259. 10.1016/j.cities.2011.02.002

[B65] Ponzini D. (2014). The values of starchitecture: Commodification of architectural design in contemporary cities. *Organiz. Aesthet.* 3 10–18.

[B66] Purcell T. Peron E. Berto R. (2001). Why do preferences differ between scene types? *Environ. Behav.* 33 93–106. 10.1177/00139160121972882

[B67] Roberts L. (1991). Current Issues in Museum Learning. *J. Museum Educat.* 16 17–18.

[B68] Roberts L. (1992). Affective Learning: Affective experience. *Visit. Stud.* 4:162.

[B69] Roe J. (2008). *The restorative power of natural and built environments.* United Kingdom: Heriot-Watt University. Doctoral dissertation.

[B70] Russell J. A. (1988). “Affective appraisals of environments,” in *Environmental aesthetics: Theory, research, and application*, ed. Nasar J. L. (Cambridge: Cambridge University Press), 120–132. 10.1017/cbo9780511571213.014

[B71] Russell J. A. (2003). Core affect and the psychological construction of emotion. *Psychol. Rev.* 110:145. 10.1037/0033-295X.110.1.145 12529060

[B72] Rybczynski W. (2002). The Bilbao Effect. *Atlant. Monthly* 290 138–142.

[B73] Scherer K. R. (2004). Which emotions can be induced by music? What are the underlying mechanisms? And how can we measure them?. *J. New Music Res.* 33 239–251. 10.1080/0929821042000317822

[B74] Schorch P. (2014). Cultural feelings and the making of meaning. *Int. J. Herit. Stud.* 20 22–35. 10.1080/13527258.2012.709194

[B75] Shusterman R. (1997). The end of aesthetic experience. *J. Aesthet. Art Critic.* 55 29–41. 10.2307/431602

[B76] Silvia P. J. (2009). Looking past pleasure: anger, confusion, disgust, pride, surprise, and other unusual aesthetic emotions. *Psychol. Aesthet. Creat. Arts* 3:48 10.1037/a0014632

[B77] Silvia P. J. (2010). Confusion and interest: The role of knowledge emotions in aesthetic experience. *Psychol. Aesthet. Creat. Arts* 4:75 10.1037/a0017081

[B78] Silvia P. J. (2012). “Human emotions and aesthetic experience: An overview of empirical aesthetics,” in *Aesthetic science: Connecting minds, brains, and experience*, Eds Edn, eds Shimamura A. P. Palmer S. E. (Oxford: Oxford University Press), 250–275. 10.1093/acprof:oso/9780199732142.003.0058

[B79] Sirefman S. (1999). Formed and forming: Contemporary museum architecture. *Daedalus* 128 297–320.

[B80] Stránský Z. Z. (1991). “The language of exhibitions,” in *The Language of Exhibitions Basic Papers, ICOFOM Study Series 19, Symposium*, ed. ICOM-International Committee for Museology (Vevey: ICOM.

[B81] Tabachnick B. G. Fidell L. S. (2013). *Using multivariate statistics*, Boston: Pearson.

[B82] Tinio P. P. Leder H. (2009). Just how stable are stable aesthetic features? Symmetry, complexity, and the jaws of massive familiarization. *Acta Psychol.* 130 241–250. 10.1016/j.actpsy.2009.01.001 19217589

[B83] Tröndle M. Wintzerith S. Wäspe R. Tschacher W. (2012). A museum for the twenty-first century: The influence of ‘sociality’ on art reception in museum space. *Museum Manag. Curat.* 27 461–486. 10.1080/09647775.2012.737615

[B84] Umiker-Sebeok J. (1994). Behavior in a museum: a semio-cognitive approach to museum consumption experiences. *Signifying Behav.* 1 52–100.

[B85] Weltzl-Fairchild A. (1995). “The museum as medium in the aesthetic response of schoolchildren,” in *Museum, Media, Message*, ed. Hooper-Greenhill E. (London: Routledge).

[B86] Zeki S. (1993). *A vision of the brain.* Oxford: Blackwell.

[B87] Zeki S. (2002). *Inner vision: An exploration of art and the brain.* Oxford: Oxford University Press.

[B88] Zeki S. (2004). The neurology of ambiguity. *Conscious. Cogn.* 13 173–196. 10.1016/j.concog.2003.10.003 14990252

